# 

**Published:** 1898-05

**Authors:** 


### CONTENTS:

### SELECTIONS.

Article I.—Pathologic Conditions of the Pharynx and Contiguous Structures During Early Childhood, Prime Factors in the Etiology of Malformed Maxillæ and Irregular Teeth, Etc. Wil iam A. Mills, D. D. S ... 1

" II.—Method of Shortening the Period of Pain During the Action of Arsenical Applications ... 5

" III.—The Drug Habit. A. W. Harlan, M. D., D. D. S ... 7

" IV.—A Clinic on a Fusible Alloy. Grant Molyneaux, D.D S. 12

" V.—Use of Screws. H. W. Arthur, D. D. S ... 16

" VI.—Some Failures Attending the Use of Weld's Chemico-Metallic Method of Filling Root Canals. Dr. H. B. Hinman ... 22

" VII.—Dentistry in the Twenties. D. D. Atkinson, D. D. S... 23

" VIII.—Amalgam ... 31

" IX.—Little Things in Great Things ... 33

### COMMUNICATED.

New Jersey State Dental Society ... 36

The National Dental Association ... 37

Meeting of American Medical Publishers' Association ... 38

The Chicago Dental Society ... 38

### MONTHLY SUMMARY.

Liquefied Air ... 39

Dishonest Dentistry ... 40

Pyorrhea Alveolaris ... 42

Caries vs. Erosion ... 42

Saucer-shaped Cavities ... 44

Opening the Bite with Cap Fillings, Preserving the Vitality of the Pulp ... 44

A Mysterious Stain ... 45

To Painlessly Cure a Corn ... 45

The Use of Non-Cohesive Gold ... 46

Immediate Investing Material ... 46

Taking Difficult Impressions ... 47

Mixing Amalgam ... 47

Dr. Evans ... 78

